# Antioxidant Activity and Total Phenolic and Flavonoid Content of Various Solvent Extracts from *In Vivo* and *In Vitro* Grown *Trifolium pratense* L. (Red Clover)

**DOI:** 10.1155/2015/643285

**Published:** 2015-05-06

**Authors:** Arash Khorasani Esmaeili, Rosna Mat Taha, Sadegh Mohajer, Behrooz Banisalam

**Affiliations:** Institute of Biological Sciences, Faculty of Science, University of Malaya, 50603 Kuala Lumpur, Malaysia

## Abstract

In the present study the extracts of* in vivo* and* in vitro* grown plants as well as callus tissue of red clover were tested for their antioxidant activities, using different extraction solvent and different antioxidant assays. The total flavonoid and phenolic contents as well as extraction yield of the extracts were also investigated to determine their correlation with the antioxidant activity of the extracts. Among all the tested extracts the highest amounts of total phenolic and total flavonoids content were found in methanol extract of* in vivo* grown plants. The antioxidant activity of tested samples followed the order* in vivo* plant extract > callus extract >* in vitro* extract. The highest reducing power, 2,2-azino-bis-(3-ethylbenzothiazoline-6-sulphonic acid) (ABTS) radical scavenging, and chelating power were found in methanol extracts of* in vivo* grown red clover, while the chloroform fraction of* in vivo* grown plants showed the highest 2,2-diphenyl-1-picrylhydrazyl (DPPH) radical scavenging, superoxide anion radical scavenging and hydrogen peroxide scavenging compared to the other tested extracts. A significant correlation was found between the antioxidant activity of extracts and their total phenolic and total flavonoid content. According to the findings, the extract of* in vitro* culture of red clover especially the callus tissue possesses a comparable antioxidant activity to the* in vivo* cultured plants' extract.

## 1. Introduction

As a member of the family Leguminosae or Fabaceae,* Trifolium pratense* L. (red clover) is a short-lived biennial plant which serves as food for livestock, but also as a health food for humans [1].

Red clover (*Trifolium pratense* L.) has high concentrations of isoflavonoids, compounds largely distributed in the Leguminosae family [2]. Many isoflavone preparations obtained from red clover are currently available as nutritional supplements [3]. Isoflavonoids are secondary metabolites divisible into isoflavones and pterocarpans. The main isoflavones in red clover are biochanin A and formononetin, both of which are found in abundance in leaves [4]. It also has antioxidant activity that may result from the presence of different flavonoids and other phenolic compounds such as phenolic acids, clovamides, and saponins. Apart from that, it has also been adopted in traditional medicine to treat whooping cough, asthma, eczema, and eye diseases [5].

As shown in recent years, natural antioxidants discovered in plants have attracted some interest due to their widely acclaimed nutritional and therapeutic values. Antioxidant properties stand to be an essential mechanism of beneficial activity of plant-derived compounds and extracts.

Ethnopharmacological surveys have shed light on the fact that the therapeutic use of even 80% of 122 plant-derived drugs may have a link with their recommendations in traditional medicine [6]. Natural antioxidants have a diversity of biochemical activities, some of which include the inhibition of reactive oxygen species (ROS) generation, direct or indirect scavenging of free radicals, and alteration of intracellular redox potential [7]. Antioxidants have functioned to inhibit apoptosis because apoptosis was at first thought to be mediated by oxidative stress [8]. It is known that many antioxidant substances have anticancer or anticarcinogenic properties [9, 10]. Epigallocatechin-3- gallate (EGCG) in green tea, for instance, has been reported to scavenge free radicals [11] and to hinder carcinogen-induced tumors in the skin, lung, forestomach, and colon of rodents [12].

Therefore, there has been undeniable evidence of interest when it comes to finding natural antioxidants from plant materials.

Studies regarding the bioactivities of various plants have assumed an important position because of the variations in the effectiveness of the plant extract with the solvent for extraction used, plant part used, the plants' age, and geographic origin. The excessive use of medicinal plants for drug formulation also puts pressure on the need for more biomass of plants which can be met with biotechnological tools like micropropagation.

Our current work aims to evaluate the antioxidant properties of the plant extracts, prepared from the aerial parts of* in vitro* and* in vivo* grown red clover. We also seek to examine the effects of extraction solvent on total phenolics, total flavonoids, and antioxidant activities of extracts from both the* in vivo* and* in vitro* grown red clover.

## 2. Materials and Methods

### 2.1. *In Vivo* Plant Samples

Seeds of* Trifolium pratense* L. were purchased from Stock Seed Farms (Murdock, US) and planted in polybags filled with coco peat, sand, and compost in the ratio of 1 : 1 : 1. The organic fertilizer was then added to each polybag and kept in the growth room (at temperature of 25 ± 1°C, 16 hours photoperiod, 40 *μ*mol/m^2^/s of photon flux density, and 60% relative humidity).

### 2.2. *In Vitro* Plant Samples

Some of the purchased seeds were surface sterilized by first placing them under the running tap water for 30 minutes and were rinsed once using sterile distilled water and soaked in 50% Clorox for 2 minutes. The seeds were then washed 5 times using sterile distilled water and soaked in 10% Clorox for one minute. Again, the seeds were rinsed 5 times using sterile distilled water and dipped in 70% ethanol for 1 minute (inside laminar flow chamber). As the final step, the seeds were rinsed 5 times using sterile distilled water and blotted on sterilized filter paper. The MS (Murashige and Skoog) medium, without the addition of any hormones, served to germinate the sterilized seeds. Cultures were stored in growth room (at 25 ± 1°C, 16 hours photoperiod, 40 *μ*mol/m^2^/s of photon flux density, and 60% relative humidity) until the grown plants are deemed suitable to be used for further investigation.

The* in vitro* regenerated red clover after six weeks of culture was used as one of the plant materials in our current study.

The callus tissue of red clover was also produced using MS medium containing 1.5 mg/L BAP (6-benzylaminopurine) and 0.5 mg/L 2,4-D (2,4-dichlorophenoxyacetic acid). The node explants of 6 weeks old* in vitro* grown plants were cultured in the media. The six-week-old callus was collected as one of the* in vitro* plant materials/samples.

### 2.3. Extract Preparation

The aerial parts of* in vivo* and* in vitro* grown plants and also the callus obtained from* in vitro* culture were collected and dried in the dark. The dried samples were powdered in an electronic blender before being used for solvent extraction. For the preparation of extract, 25 g of fine powder was extracted with 100 mL of 95% methanol at room temperature for 48 hours. The extracts were filtrated through Whatman number 1 and combined and this was followed by a concentration using rotary evaporator under pressure that was reduced at 45°C. The filtrate obtained was suspended in distilled water (25 mL) and n-hexane (50 mL) was added to it, where the mixture was shaken well and the layers were enabled to be separated for 6 h. After separation of n-hexane layer again 50 mL of n-hexane was added to get the n-hexane fraction. In a similar manner, the protocol was repeated with the rest of the solvents (ethyl acetate and chloroform). Each fraction obtained was dried using a rotary evaporator [13]. The dry extract obtained with each solvent was weighed and the percentage yield was expressed in terms of the air-dried weight of plant materials. Samples were stored in an airtight container at –20°C until it was time to conduct further analysis.

### 2.4. Determination of Antioxidant Activities

#### 2.4.1. Total Phenolic Content

The total phenolic content of the obtained extracts was spectrometrically analyzed in adherence to the Folin-Ciocalteu method [14]. In short, 100 *μ*L of each extract (dissolved in methanol) or gallic acid standard solution was mixed with 2 mL of 2% (w/v) sodium carbonate solution. The mixture was then incubated for 5 minutes, and afterwards 100 *μ*L of Folin-Ciocalteu reagent was added. After being left for 30 min at room temperature for color development, absorbance was measured at 750 nm using a spectrophotometer. Results are expressed as mg gallic acid equivalents (GAE) per gram dry matter of sample.

#### 2.4.2. Total Flavonoid Content

Total flavonoid content was ascertained based on the method by Zhishen et al. [15]. In brief, 50 mg of each fraction was dissolved in 10 mL of 80% aqueous methanol and filtered through Whatman filter paper number 42 (125 mm). In a 10 mL test tube, 300 *μ*L of each extract, 3.4 mL of 30% methanol, 150 *μ*L of 0.5 M NaNO_2_, and 150 *μ*L of 0.3 M AlCl_3_·6H_2_O were added and mixed, followed by 5 min incubation and the addition of 1 mL of NaOH (1 M). Absorbance was measured at 510 nm with a spectrophotometer. The standard curve for total flavonoids was made using rutin standard solution (0–100 mg/L) using the same aforementioned procedure. The total flavonoid content was shown as milligram of rutin equivalents (CTE) per gram dry matter of extract.

#### 2.4.3. Antioxidant Assays

Each sample was dissolved in 95% methanol at a concentration of 1 mg/mL and then diluted in order to prepare the series concentrations for antioxidant assays. Reference chemicals were used for comparative purposes in all assays.

#### 2.4.4. DPPH Radical Scavenging Activity Assay

The DPPH radical scavenging assay was conducted following the method of Zhu et al. [16]. In brief, 2 mL of DPPH solution (0.1 mM, in methanol) was blended with 2 mL of the samples at varying concentrations (50, 100, 150, 200, 250, and 300 *μ*g/mL). As the next stage, the reaction mixture was shaken and incubated in the dark at room temperature for 30 min, and the absorbance was read at 517 nm against the blank. Ascorbic acid and rutin standard as positive controls were prepared in a similar manner, as for the test group except for the antioxidant solution's replacement. The inhibition of the DPPH radical by the sample was calculated based on the formula below:

(1) DPPH  scavenging  activity%=absorbance  of  control−absorbance  of  sampleabsorbance  of  control×100.

#### 2.4.5. Superoxide Anion Radical Scavenging Assay

The assay for superoxide anion radical scavenging activity leaned on a riboflavin-light-NBT system [17]. The reaction mixture had 0.5 mL of phosphate buffer (50 mM, pH 7.6), 0.3 mL riboflavin (50 mM), 0.25 mL phenazine methosulphate (PMS) (20 mM), and 0.1 mL nitro blue tetrazolium (NBT) (0.5 mM), before 1 mL sample solution was added at varying concentrations (50, 100, 150, 200, 250, and 300 *μ*g/mL). Reaction began as the reaction mixture was illuminated with different concentrations of the extracts using a fluorescent lamp. After 20 min of incubation, the absorbance was measured at 560 nm. Ascorbic acid was used as standard. The percentage of inhibition of superoxide anion generation was calculated based on the following formula:

(2) Scavenging  activity%=1−absorbance  of  sampleabsorbance  of  control×100.

#### 2.4.6. ABTS Radical Scavenging Assay

The ABTS radical scavenging assay was performed adhering to the method of Re et al. [18] with slight modification. The ABTS radical was generated through the oxidation of ABTS with potassium persulfate. In brief, the ABTS solution (7 mM) had reacted with potassium persulfate (2.45 mM) solution and was stored in the dark for 12–16 h to produce a dark coloured solution containing ABTS radical cation. Before being used in the assay, the ABTS radical cation was diluted with 50% methanol for an initial absorbance of about 0.70 (±0.02) at 745 nm, with the temperature control fixed as 30°C. Free radical scavenging activity was evaluated by mixing 3 mL of ABTS working standard with 300 *μ*L of test sample (50, 100, 150, 200, 300, 400, and 500 *μ*g/mL) in a microcuvette. The decrease in the absorbance was measured at the exact time of 1 min after mixing the solution until it reached 6 min. The final absorbance was noted then. The inhibition percentage was calculated based on the following formula:

(3) Scavenging  effect%=absorbance  of  control−absorbance  of  sampleabsorbance  of  control×100.

#### 2.4.7. Hydrogen Peroxide Scavenging Activity

The capability of scavenging hydrogen peroxide by the extract was determined based on the method of Ruch et al. [19]. A hydrogen peroxide solution (2 mM) was prepared in 50 mM phosphate buffer (pH 7.4). Aliquots (0.1 mL) of the extracted sample (different concentration of 50, 100, 150, 200, 250, and 300 *μ*g/mL) were transferred into the test tubes and their volumes were made up to 0.4 mL with 50 mM phosphate buffer (pH 7.4). After adding 0.6 mL hydrogen peroxide solution, tubes were vortexed and the absorbance of the hydrogen peroxide at 230 nm was determined after 10 min, against a blank. The abilities to scavenge the hydrogen peroxide were calculated based on the following equation:

(4) Hydrogen  peroxide  scavenging  activity=1−absorbance  of  sampleabsorbance  of  control×100.

#### 2.4.8. Reducing Power Assay

The reducing powers of the samples were determined following the method of Atmani et al. [20]. Two millilitres of each extract solution (300 *μ*g/mL) was mixed with 2 mL of phosphate buffered saline (0.2 M, pH 6.6) and 2 mL of potassium ferrocyanate (10 mg/mL). The incubation for this mixture was set at 50°C for 20 min. In the next stage, 2 mL of trichloroacetic acid (100 mg/L) was added to the mixture. In a test tube, a volume of 2 mL from each of the above mixtures was mixed with 2 mL of distilled water and 0.4 mL of 0.1% (w/v) ferric chloride. The absorbance was measured at 700 nm after reaction was started for 10 minutes. The increased absorbance of the reaction mixture suggested that the reducing power was high.

#### 2.4.9. Chelating Power

The ability of the extract to chelate iron (II) was estimated based on the method of Dinis et al. [21] with minimal modification. Various sample solutions (50–300 *μ*g/mL) were prepared with dissolving the extracts in the methanol. An aliquot of each sample (200 *μ*L) was mixed with 100 *μ*L of FeCl_2_·2H_2_O (2 mM) and 900 *μ*L of methanol. After 5 min incubation, an initial reaction was fuelled by the addition of 400 *μ*L of ferrozine (5 mM). After 10 min incubation, the absorbance at 562 nm was recorded. The percentage of the chelating activity was calculated based on the following equation:

(5) Chelating  activity%=absorbance  of  control−absorbance  of  sampleabsorbance  of  control×100.

### 2.5. Statistical Analysis

All assays were carried out in triplicate and their results were expressed as mean ± standard deviation. The EC_50_ (half-maximal effective concentration) of various fractions for different antioxidant assays were analysed using ANOVA test with least significant difference (LSD) *P* < 0.05 as a level of significance. Experimental results were examined further for Pearson correlation coefficient of phenolic and flavonoids with different antioxidants assays and its significance was tested using Student's* t*-test (*P* < 0.05).

## 3. Results and Discussion

### 3.1. Total Phenolic, Total Flavonoid, and Extraction Yield

Good extraction methods prove to be a crucial step for getting extracts with acceptable yields and strong antioxidant activity [22]. The percentage yields of the methanol extract and different fractions of* in vivo* and* in vitro* grown plants as well as the callus tissue of red clover are shown in Table 1. The extraction yield of these samples came in a range from 2.35 ± 0.5% to 13.89 ± 0.54%. The results showed that the methanol extraction of* in vivo* grown plants had illustrated the highest amount of extraction yield, and conversely the extraction yield of* in vitro* grown plants with ethyl acetate was prominently lower (*P* < 0.05) when compared to that of the other samples. Being plant secondary metabolites, the phenolics or polyphenols are very important judging from the virtue of their antioxidant activities by chelating redox-active metal ions, inactivating lipid free radical chains, and avoiding the hydroperoxide conversions into reactive oxyradicals. The total phenolic contents of the extracts, expressed as gallic acid equivalents, varied from 46.88 ± 1.07 mg GAE/g for the methanol extract of* in vivo* grown plants to 16.9 ± 1.19 mg GAE/g for the ethyl acetate extract of* in vitro* grown plants (Table 1). In all* in vivo* and* in vitro* plants and also callus tissue, the methanolic extract had illustrated the highest total content of phenolic, whereas the content obtained with ethyl acetate was much lower (*P* < 0.05), which is similar to the reports compiled by Sahreen et al. [23] and Ao et al. [24]. A possible justification would be due to the formation of complexes by a part of phenolic compounds with carbohydrates and proteins, which are more extractable in methanol than in other solvents that have been emphasized in this study. Our results do not deviate from those of Parsaeimehr et al. [25] who also found that wild plant, compared to the callus tissue, showed higher extracting phenolic components with regard to some medicinal plants. The rich-flavonoid plants could manifest themselves as good sources of antioxidants that would assist in the enhancement of the overall antioxidant capacity of an organism and protection against lipid peroxidation [26]. The content of total flavonoids is expressed as mg of rutin equivalents per g of dry sample that ranged from 6.11 ± 0.79 to 26.61 ± 0.92, the amounts of which were comparable with results verified in the literatures for other extracts produced [24, 27]. The methanol extract of* in vivo* grown red clover significantly contained (*P* < 0.05) higher flavonoids concentration as compared to other samples involved. The lowest flavonoid value in this study was recorded in ethyl acetate fraction of* in vitro* grown plants (6.11 ± 0.79 mg CTE/g dry sample). The total flavonoid content results were entirely synchronous with those of the total phenolic. It was successfully shown that samples with high level of phenolic content also contain flavonoids in great amount. The rich-flavonoid plants could be a good antioxidant source that would help increase the overall antioxidant capacity of an organism and guard it against lipid peroxidation [26].

### 3.2. DPPH Radical Scavenging Activity

**Figure 1:**
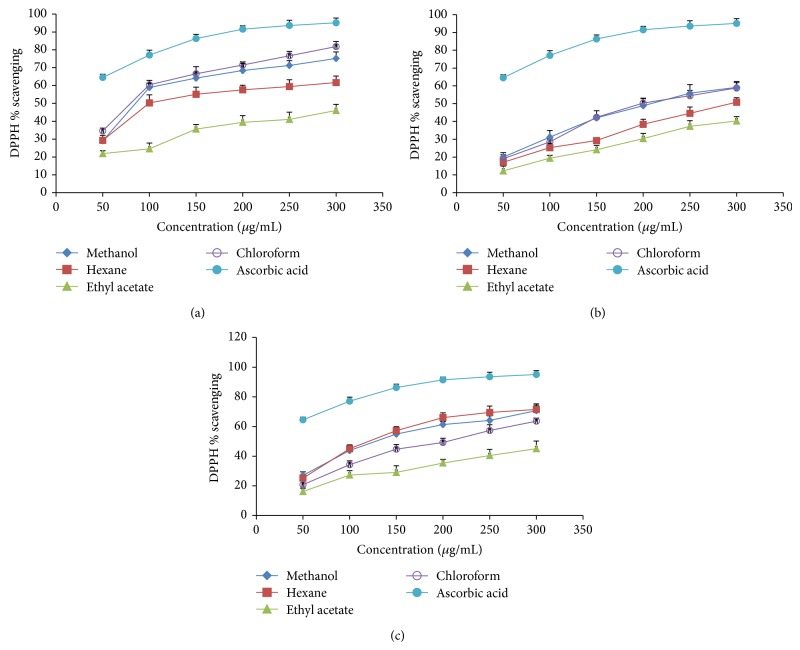
DPPH radical scavenging activity of different extracts from the methanol extract of* Trifolium pratense* by different solvent at different concentration. (a)* In vivo* grown plants, (b)* in vitro* grown plants, and (c) callus tissue. Each value is represented as mean ± SD (*n* = 3).

2,2-Diphenyl-1-picrylhydrazyl radical is a stable organic free radical with an absorption band at 517 nm. It loses this absorption when it accepts an electron or a free radical species, resulting in a visually noticeable discoloration from purple to yellow. It can incorporate many samples in a short time span and is vulnerable enough to distinguish active ingredients at low concentrations [28].  Figure 1 highlights the DPPH radical scavenging ability of* in vivo*,* in vitro,* and callus samples with different extraction solvents. The differential scavenging activities of the extract against the DPPH system that has been observed could be explained by the presence of different compounds in the fractions. The chloroform fraction of* in vivo* grown plants showed a reading of the lowest EC_50_ of DPPH radical scavenging (81.04 ± 2.33 *μ*g/mL), while the highest EC_50_ belonged to the ethyl acetate fraction of* in vitro* grown plants (≫300 *μ*g/mL) (Table 2). Although the DPPH radical scavenging activities of the fractions were less (*P* < 0.05) than those of the ascorbic acid, the study had made a disclosure that* in vivo* grown plants and callus tissue of red clover have free radical scavengers or inhibitors, possibly acting as primary antioxidants more than* in vitro* grown red clover. There was an observation on a similar trend in a study of the antioxidant activity of the* Artemisia judaica* L. extract [29].

### 3.3. Superoxide Radical Scavenging Activity

**Figure 2:**
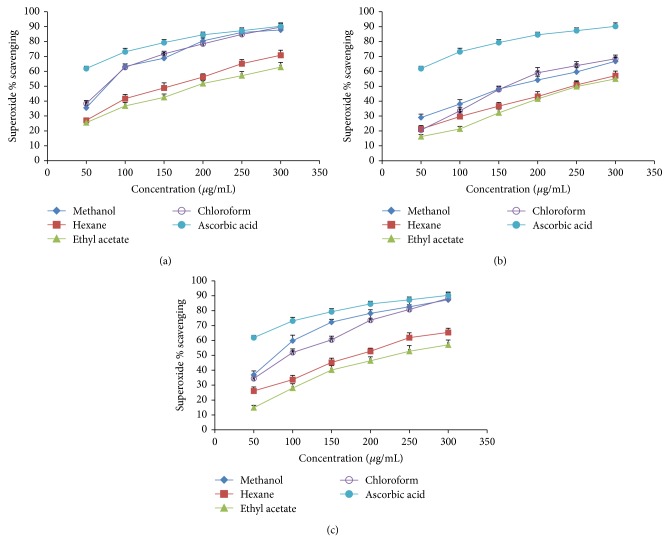
Superoxide radical scavenging activity of different extracts from the methanol extract of* Trifolium pratense* by different solvent at different concentration. (a)* In vivo* grown plants, (b)* in vitro* grown plants, and (c) callus tissue. Each value is represented as mean ± SD (*n* = 3).

As it is a reactive oxygen species, superoxide has some damaging properties that can be imposed to the cells and DNA and subsequently invites various diseases. Thus, a proposal has been established to gauge the comparative interceptive ability of the antioxidant extracts to scavenge the superoxide radical [30]. The superoxide radical scavenging effect of these varying fractions of methanol extract of* in vivo *and* in vitro *grown plants as well as the callus tissue of red clover was drawn in comparison with the same doses of ascorbic acid in a range from 50 to 300 *μ*g/mL as shown in Figure 2. The results had suggested that the lowest EC_50_ value (70.69 ± 1.76 *μ*g/mL) belongs to the chloroform fraction of* in vivo* samples, whereas the highest value (260.01 ± 1.46 *μ*g/mL) belongs to the ethyl acetate fraction of* in vitro* samples (Table 3). In a dose dependent manner, all of the fractions had scavenging activities on the superoxide radicals. Nonetheless, when compared to the ascorbic acid, the superoxide scavenging activities of the extracts were found to be significantly lower (*P* < 0.05). This could be due to the presence of flavonoids and other antioxidants in the extract.

### 3.4. ABTS Radical Scavenging Activity

The ABTS radical cation decolourisation test is another widely established approach adopted to evaluate antioxidant activity. Colour reduction shows the decrease of ABTS radical [31]. The samples' ABTS radical scavenging capability can be ranked as follows:* in vivo *> callus >* in vitro* samples. The methanol extract of* in vivo* grown samples had demonstrated the highest radical scavenging activity when it reacted with the ABTS radicals. By contrast, the ethyl acetate fraction of* in vivo*,* in vitro,* and callus samples and also the n-hexane fraction of* in vitro* samples did not illustrate any leveling effect at the highest concentration, but it was a fact that their radical scavenging effects were much less (*P* < 0.05) than that of the other extracts tested (Table 4). Significant differences among the EC_50_ values of all the fractions and ascorbic acid had also been noted (*P* < 0.05).

### 3.5. Hydrogen Peroxide

While hydrogen peroxide itself is not very reactive, it can sometimes be poisonous to cells, since it may trigger the rise of hydroxyl radicals inside the cell [32]. Extracts from* in vivo* and* in vitro* grown plants as well as callus tissue of red clover had the capability to scavenge hydrogen peroxide in a concentration dependent manner (50–300 *μ*g/mL). The hydrogen peroxide scavenging activities of* in vivo* grown samples were proven to be more effective (*P* < 0.05) than the* in vitro* grown and callus samples, as revealed by the comparison with the EC_50_ values. The chloroform fraction and methanol extract were found to be more efficient (*P* < 0.05) than those of the n-hexane and ethyl acetate fractions. EC_50_ values of all the extracts, in scavenging abilities on hydrogen peroxide, were remarkably different (*P* < 0.05) from the EC_50_ value that had been obtained for ascorbic acid (Table 5).

### 3.6. Chelating Activity

Ability to chelate or deactivate transition metals, which in turn has the ability to catalyze hydroperoxide decomposition and Fenton-type reactions, is a vital mechanism of antioxidant activity. It was thus considered of importance to screen the iron (II) chelating ability of extracts [27]. All the fractions had ferrous ion chelating activity but they were remarkably low (*P* < 0.05) in comparison to catechin. The sequence for the chelating power was* in vivo* grown samples > callus tissue of samples >* in vitro* grown samples. The highest chelating power was shown by the methanol extract of* in vivo* grown plants with the EC_50_ value of 49.11 ± 0.97 *μ*g/mL, whilst the lowest was found in ethyl acetate fraction of* in vitro* grown samples with the EC_50_ value of 183.44 ± 2.48 *μ*g/mL (Table 6). The ion chelating data with different measurements of concentrations (50–300 *μ*g/mL) suggested that ferrous ion chelating effects of all the fractions of* in vivo* and* in vitro* grown plants as well as callus tissue of red clover would be rather advantageous to offer protection against oxidative damage.

### 3.7. Reducing Power Activity

The reducing power of the extract, which potentially serves as a significant reflection of the antioxidant activity, was ascertained using a modified Fe^3+^ to Fe^2+^ reduction assay, whereby the colour of the test solution, which was yellow, transforms to various hues of green and blue, based on the extent of the reducing power of the samples. The presence of the antioxidants in the samples leads to Fe^3+^/ferricyanide complex reduced to the Fe^2+^ form and Fe^2+^ can be monitored through the measurement of the formation of Perl's Prussian blue at 700 nm [33].

In the reducing power assay, the presence of reductants (antioxidants) in the fractions would bring about the reduction of Fe^+3^/ferricyanide complex to the ferrous form by giving away an electron. Increasing the absorbance at 700 nm implies an increase in its ability to reduce. The sequence for this reducing power was as follows:* in vivo* samples > callus tissue >* in vitro *samples of red clover. Some degrees of electron-donating capacity were found in all examined extracts. Among the examined extracts the methanol extract of* in vivo* samples showed the highest reducing power with the absorbance of 1.05 ± 0.07 at 700 nm, so this extract could act as electron donors and also could convert free radicals to more stable products [34], although, in comparison with the positive control (ascorbic acid), it is significantly lower (Table 7).

### 3.8. Correlation with Antioxidant Activities and Phytochemical Contents

Through the correlation analysis for phytochemical contents with EC_50_ values of radical scavenging activity and antioxidant ability of the extract of red clover and its various soluble fractions, the phenolic and flavonoid contents had exhibited excellent association with DPPH, superoxide, ABTS radical scavenging activities, and reducing power of* in vivo*,* in vitro,* and callus samples (Tables 8, 9, and 10). Erkan et al. [35] reported a close correlation between radical scavenging activity and total phenolic content of extract from various natural sources. Moreover, for the* in vivo* samples, EC_50_ of hydrogen peroxide presented an important correlation with phenolics while nonsignificant with flavonoids, whereas for the* in vitro* samples hydrogen peroxide demonstrated a remarkable correlation with both phenolics and flavonoids. Hydrogen peroxide of callus samples did not shed light on any correlation with phenolics and flavonoids. Chelating power of* in vivo* samples pointed to a significant correlation with flavonoids but nonsignificant correlation with phenolics while for the callus samples it was found with both phenolics and flavonoids and for the* in vitro* samples chelating power did not point to any correlation with phenolics and flavonoids. Our results are consistent with those found by Sahreen et al. [23] who reported that there is existence of a strong relationship between phytochemical contents and DPPH and superoxide and ABTS radical scavenging.

## 4. Conclusion

The present work shows the antioxidant effects of* in vivo* and* in vitro* grown plants and callus tissues of red clover. The results also suggested a considerable value in terms of the antioxidant activities of methanol extract and chloroform fraction of aerial parts of the* in vivo* grown red clover. The fraction's activity is due to the phenolic and flavonoid contents. It is further suggested by our results that the extract of red clover can be adopted as an effective and safe antioxidant source, despite the fact that the antioxidant activities of methanol extract and chloroform fraction of red clover were lower than that ascorbic acid and rutin as positive controls. It is safe to sum up that red clover is consumed as a traditional medicine and food stuff in various parts of the world and that it can be used as an achievable source of natural antioxidants with consequent beneficial properties for health. There is indeed a pressing need to make available new plant-derived bioactive molecules; thus, red clover may be a great natural source for the establishment of new drugs.

In earlier reports, such properties have been named as essential for the extracts of red clover [36]. Based on the results obtained, the extracts of callus tissue had demonstrated a strong antioxidant activity but the extract of aerial parts of* in vitro* cultured red clover had demonstrated a lower antioxidant activity. Matsingou et al. [37] have reported that the differences in antioxidant properties of plants in varying biological systems may be explained by the presence of different substrates and also by the variable nature of product produced by the reaction system. It was observed that, due to a variety of antioxidant compounds present in red clover, the activity of the extract was attributed to the samples extraction method and the assay method.

Our primary finding of this work was that extract of* in vitro* culture of red clover, especially the callus tissue, possesses an antioxidant activity comparable to the* in vivo* cultured plants' extract. It can be concluded that* in vitro* cultured plants are able to produce and accumulate many medicinally valuable secondary metabolites.

## Figures and Tables

**Table 1:** Total phenolic, total flavonoid, and extraction yield of methanol extract and soluble fractions of *in  vivo* and *in vitro* grown plants (aerial parts) and also callus tissue of *Trifolium pretense. *

Plant extract	Total phenolic (mg gallic acid/g dry sample)	Total flavonoid (mg rutin equivalent/g dry sample)	Extraction yield (%)
*In vivo *			
Methanol extract	46.88 ± 1.07^a^	26.61 ± 0.92^a^	13.89 ± 0.54^a^
n-Hexane fraction	30.52 ± 0.72^cd^	16.06 ± 1.58^d^	10.79 ± 0.59^c^
Ethyl acetate fraction	27.57 ± 0.49^e^	11.71 ± 1.43^fg^	6.39 ± 1.12^e^
Chloroform fraction	40.17 ± 0.88^b^	19.56 ± 1.11^b^	12.23 ± 0.54^b^
*In vitro *			
Methanol extract	31.94 ± 1.68^c^	13.03 ± 0.79^ef^	10.84 ± 0.72^c^
n-Hexane fraction	20.06 ± 0.41^g^	8.23 ± 0.96^h^	5.20 ± 0.54^f^
Ethyl acetate fraction	16.90 ± 1.19^h^	6.11 ± 0.79^i^	2.35 ± 0.5^g^
Chloroform fraction	25.43 ± 0.87^f^	10.39 ± 0.79^g^	7.18 ± 0.82^e^
Callus			
Methanol extract	40.82 ± 1.5^b^	17.84 ± 0.53^c^	11.36 ± 0.55^bc^
n-Hexane fraction	31.77 ± 1.14^c^	12.70 ± 0.94^ef^	6.13 ± 0.34^ef^
Ethyl acetate fraction	19.23 ± 0.66^g^	7.21 ± 0.74^hi^	2.98 ± 0.66^g^
Chloroform fraction	29.25 ± 1.62^de^	14.02 ± 1.08^e^	8.70 ± 0.52^d^

Each value is represented as mean ± SD (*n* = 3).

Means in the same column not sharing the same letters are significantly different at (Duncan) *P* < 0.05.

**Table 2:** DPPH radical scavenging activity of methanol extract and soluble fractions of *in vivo* and *in vitro* grown plants and callus tissue of red clover (*Trifolium pratense*).

Plant extract	EC_50_ of DPPH radical scavenging activity (*µ*g/mL)		
	*In vivo *	*In vitro *	Callus
Methanol extract	94.25 ± 1.15^c∗^	205.47 ± 2.90^b∗∗∗^	128.42 ± 2.40^*c*∗∗^
n-Hexane fraction	131.42 ± 3.20^d∗∗^	291.95 ± 2.98^c∗∗∗^	120.74 ± 3.45^b∗^
Ethyl acetate fraction	>300^e∗^	≫300^d∗∗∗^	>300^e∗∗^
Chloroform fraction	81.04 ± 2.33^b∗^	208.86 ± 2.20^b∗∗∗^	155.17 ± 2.80^d∗∗^
Ascorbic acid	21.17 ± 0.76^a^	21.17 ± 0.76^a^	21.17 ± 0.76^a^

Each value in the table is represented as mean ± SD (*n* = 3).

Means not sharing the same symbols (∗, ∗∗, and ∗∗∗) are significantly different (Duncan) at *P* < 0.05 in the same row.

Means not sharing the same letters are significantly different (Duncan) at *P* < 0.05 in the same column.

**Table 3:** Superoxide anion activity of methanol extract and soluble fractions of *in vivo* and *in vitro* grown plants and callus tissue of red clover (*Trifolium pratense*).

Plant extract	EC_50_ of superoxide anion activity (*µ*g/mL)		
	*In vivo *	*In vitro *	Callus
Methanol extract	74.68 ± 1.59^c∗^	179.77 ± 1.60^c∗∗^	74.36 ± 1.42^b∗^
n-Hexane fraction	139.83 ± 2.40^d∗^	246.59 ± 2.09^d∗∗∗^	190.06 ± 2.56^d∗∗^
Ethyl acetate fraction	201.55 ± 1.94^e∗^	260.01 ± 1.46^e∗∗∗^	228.41 ± 1.52^e∗∗^
Chloroform fraction	70.69 ± 1.76^b∗^	155.91 ± 1.73^b∗∗∗^	104.32 ± 1.45^c∗∗^
Ascorbic acid	23.44 ± 0.84^a^	23.44 ± 0.84^a^	23.44 ± 0.84^a^

Each value in the table is represented as mean ± SD (*n* = 3).

Means not sharing the same symbols (∗, ∗∗, and ∗∗∗) are significantly different (Duncan) at *P* < 0.05 in the same row.

Means not sharing the same letters are significantly different (Duncan) at *P* < 0.05 in the same column.

**Table 4:** ABTS radical scavenging of methanol extract and soluble fractions of *in vivo* and *in vitro* grown plants and callus tissue of red clover (*Trifolium pratense*).

Plant extract	EC_50_ of ABTS (*µ*g/mL)		
	*In vivo *	*In vitro *	Callus
Methanol extract	111.84 ± 1.46^b∗^	351.46 ± 2.02^c∗∗∗^	164.32 ± 1.61^b∗∗^
n-Hexane fraction	210.76 ± 1.71^d∗^	>500^d∗∗∗^	225.63 ± 2.05^d∗∗^
Ethyl acetate fraction	>500^e∗^	≫500^e∗∗∗^	>500^e∗∗^
Chloroform fraction	122.34 ± 2.29^c∗^	297.68 ± 1.68^b∗∗∗^	172.18 ± 1.55^c∗∗^
Ascorbic acid	34.67 ± 0.53^a^	34.67 ± 0.53^a^	34.67 ± 0.53^a^

Each value in the table is represented as mean ± SD (*n* = 3).

Means not sharing the same symbols (∗, ∗∗, and ∗∗∗) are significantly different (Duncan) at *P* < 0.05 in the same row.

Means not sharing the same letters are significantly different (Duncan) at *P* < 0.05 in the same column.

**Table 5:** Hydrogen peroxide scavenging of methanol extract and soluble fractions of *in vivo* and *in vitro* grown plants and callus tissue of red clover (*Trifolium pratense*).

Plant extract	EC_50_ of hydrogen peroxide (*µ*g/mL)		
	*In vivo *	*In vitro *	Callus
Methanol extract	103.44 ± 1.47^c∗^	219.86 ± 1.57^b∗∗∗^	141.74 ± 1.93^c∗∗^
n-Hexane fraction	270.56 ± 1.72^e∗^	≫300^e∗∗∗^	287.63 ± 1.59^e∗∗^
Ethyl acetate fraction	138.16 ± 1.93^d∗∗^	>300^d∗∗∗^	132.26 ± 2.03^b∗^
Chloroform fraction	88.35 ± 1.27^b∗^	231.49 ± 2.02^c∗∗∗^	146.31 ± 1.67^d∗∗^
Ascorbic acid	26.84 ± 0.83^a^	26.84 ± 0.83^a^	26.84 ± 0.83^a^

Each value in the table is represented as mean ± SD (*n* = 3).

Means not sharing the same symbols (∗, ∗∗, and ∗∗∗) are significantly different (Duncan) at *P* < 0.05 in the same row.

Means not sharing the same letters are significantly different (Duncan) at *P* < 0.05 in the same column.

**Table 6:** Chelating activity of methanol extract and soluble fractions of *in vivo* and *in vitro* grown plants and callus tissue of red clover (*Trifolium pratense*).

Plant extract	EC_50_ of chelating power (*µ*g/mL)		
	*In vivo *	*In vitro *	Callus
Methanol extract	49.11 ± 0.97^b∗^	142.87 ± 1.71^c∗∗∗^	72.56 ± 1.16^d∗∗^
n-Hexane fraction	52.33 ± 1.97^c∗^	131.84 ± 2.05^b∗∗∗^	67.52 ± 2.15^c∗∗^
Ethyl acetate fraction	105.67 ± 1.56^e∗^	183.44 ± 2.48^e∗∗∗^	118.23 ± 2.02^e∗∗^
Chloroform fraction	86.32 ± 1.89^d∗∗^	161.75 ± 1.36^d∗∗∗^	60.17 ± 1.42^b∗^
Catechin	22.76 ± 0.37^a^	22.76 ± 0.37^a^	22.76 ± 0.37^a^

Each value in the table is represented as mean ± SD (*n* = 3).

Means not sharing the same symbols (∗, ∗∗, and ∗∗∗) are significantly different (Duncan) at *P* < 0.05 in the same row.

Means not sharing the same letters are significantly different (Duncan) at *P* < 0.05 in the same column.

**Table 7:** Reducing power of methanol extract and soluble fraction (300 *μ*g/mL) of *in vivo* and *in vitro* grown plants and callus tissue of red clover (*Trifolium pratense*).

Plant extract	Reducing power absorbance at 700 nm		
	*In vivo *	*In vitro *	Callus
Methanol extract	1.05 ± 0.07^b∗^	0.72 ± 0.03^b∗∗∗^	0.82 ± 0.03^c∗∗^
n-Hexane fraction	0.88 ± 0.04^c∗^	0.52 ± 0.04^c∗∗∗^	0.65 ± 0.02^d∗∗^
Ethyl acetate fraction	0.59 ± 0.03^d∗^	0.38 ± 0.01^d∗∗∗^	0.52 ± 0.03^e∗∗^
Chloroform fraction	0.95 ± 0.03^c∗^	0.71 ± 0.04^b∗∗^	0.91 ± 0.03^b∗^
Ascorbic acid	2.45 ± 0.03^a^	2.45 ± 0.03^a^	2.45 ± 0.03^a^

Each value in the table is represented as mean ± SD (*n* = 3).

Means not sharing the same symbols (∗, ∗∗, and ∗∗∗) are significantly different (Duncan) at *P* < 0.05 in the same row.

Means not sharing the same letters are significantly different (Duncan) at *P* < 0.05 in the same column.

**Table 8:** Correlation between the antioxidant activity and total phenolic and flavonoid of the extract of *in vivo* grown *Trifolium  pretense*.

Assays	Correlation *R* ^2^	
	Total flavonoid	Total phenolic
DPPH radical scavenging activity	0.759^∗∗^	0.745^∗∗^
Superoxide radical scavenging activity	0.856^∗∗^	0.903^∗∗^
ABTS radical scavenging ability	0.814^∗∗^	0.801^∗∗^
Hydrogen peroxide radical scavenging activity	0.414	0.598^∗^
Chelating power	0.670^∗^	0.500
Reducing power	0.884^∗∗^	0.861^∗∗^

^∗∗^Correlation is significant at the 0.01 level (2-tailed).

^∗^Correlation is significant at the 0.05 level (2-tailed).

**Table 9:** Correlation between the antioxidant activity and total phenolic and flavonoid of the extract of *in vitro* grown *Trifolium  pretense*.

Assays	Correlation *R* ^2^	
	Total flavonoid	Total phenolic
DPPH radical scavenging activity	0.893^∗∗^	0.876^∗∗^
Superoxide radical scavenging activity	0.793^∗∗^	0.806^∗∗^
ABTS radical scavenging ability	0.825^∗∗^	0.827^∗∗^
Hydrogen peroxide radical scavenging activity	0.784^∗∗^	0.833^∗∗^
Chelating power	0.502	0.441
Reducing power	0.862^∗∗^	0.862^∗∗^

^∗∗^Correlation is significant at the 0.01 level (2-tailed).

**Table 10:** Correlation between the antioxidant activity and total phenolic and flavonoid of the extract of callus tissue of *Trifolium  pretense*.

Assays	Correlation *R* ^2^	
	Total flavonoid	Total phenolic
DPPH radical scavenging activity	0.849^∗∗^	0.822^∗∗^
Superoxide radical scavenging activity	0.912^∗∗^	0.917^∗∗^
ABTS radical scavenging ability	0.903^∗∗^	0.876^∗∗^
Hydrogen peroxide radical scavenging activity	0.023	0.020
Chelating power	0.775^∗∗^	0.734^∗∗^
Reducing power	0.777^∗∗^	0.753^∗∗^

^∗∗^Correlation is significant at the 0.01 level (2-tailed).
